# Alleviation of Residual Vibrations in Hard-Magnetic Soft Actuators Using a Command-Shaping Scheme

**DOI:** 10.3390/polym14153037

**Published:** 2022-07-27

**Authors:** Naresh Nagal, Shikhar Srivastava, Chandan Pandey, Ankur Gupta, Atul Kumar Sharma

**Affiliations:** Department of Mechanical Engineering, Indian Institute of Technology Jodhpur, Jodhpur 342037, Rajasthan, India; nagal.1@iitj.ac.in (N.N.); srivastava.21@iitj.ac.in (S.S.); jscpandey@iitj.ac.in (C.P.); ankurgupta@iitj.ac.in (A.G.)

**Keywords:** magneto-active elastomers, hard-magnetic soft materials, hyperelasticity, nonlinear vibrations, vibration control, open-loop control

## Abstract

Hard-magnetic soft materials belong to a class of the highly deformable *magneto-active elastomer* family of smart materials and provide a promising technology for flexible electronics, soft robots, and functional metamaterials. When hard-magnetic soft actuators are driven by a multiple-step input signal (Heaviside magnetic field signal), the residual oscillations exhibited by the actuator about equilibrium positions may limit their performance and accuracy in practical applications. This work aims at developing a command-shaping scheme for alleviating residual vibrations in a magnetically driven planar hard-magnetic soft actuator. The control scheme is based on the balance of magnetic and elastic forces at a critical point in an oscillation cycle. The equation governing the dynamics of the actuator is devised using the Euler–Lagrange equation. The constitutive behaviour of the hard-magnetic soft material is modeled using the Gent model of hyperelasticity, which accounts for the strain-stiffening effects. The dynamic response of the actuator under a step input signal is obtained by numerically solving the devised dynamic governing equation using *MATLAB ODE solver*. To demonstrate the applicability of the developed command-shaping scheme, a thorough investigation showing the effect of various parameters such as material damping, the sequence of desired equilibrium positions, and polymer chain extensibility on the performance of the proposed scheme is performed. The designed control scheme is found to be effective in controlling the motion of the hard-magnetic soft actuator at any desired equilibrium position. The present study can find its potential application in the design and development of an open-loop controller for hard-magnetic soft actuators.

## 1. Introduction

Soft active materials undergo various morphological changes and large deformations under externally applied mechanical [1,2], heat [3], light [4], magnetic [5,6], electric [7,8,9,10], humidity [11] and/or solvent [12] fields. Some soft active materials include dielectric elastomers [13,14], hydrogels [15,16], liquid crystal elastomers [17,18], magneto-active elastomers [19,20,21,22,23], etc. In the recent years, these active materials have become greatly attractive due to their possible uses in the fields of soft robotics [24,25,26], flexible electronic devices [27], biomedical devices [28,29], wearable devices [30,31], soft actuators and sensors [32,33,34], among many others. In comparison to other soft active materials, magneto-active elastomers are efficient when the actuation is needed in a closed space due to the ability of a magnetic field to penetrate in a wide range of materials [35]. Hard-magnetic soft materials, a new class of materials belonging to the family of magneto-active elastomers, fabricated by embedding hard magnetic particles (like hard samarium-cobalt, ferrite, NdFeB, and many more) into soft silicon elastomers with 3D printing technology, have attracted tremendous research interest recently because of their remarkable properties, such as their fast reversible response, remote external stimuli, flexibility, ability to show programmable and complex shape transformations in low magnetic fields, etc. [36,37,38,39]. The potential applications include but are not limited to soft robotics [40], functional meta-materials/structures [41,42], and actuators [43].

For the design and development of devices and structures that are based on the promising hard-magnetic soft materials, it is crucial to understand the underlying mechanics and fundamentals of hard-magnetic soft materials. In this regard, recently, Zhao et al. [44] developed a finite deformation and nonlinear finite element framework for understanding the mechanics of ideal hard-magnetic soft materials. The developed finite element framework (implemented into the commercial software *ABAQUS* using *UEL*) was exploited to simulate the magneto-mechanical behaviour of hard-magnetic soft planar actuators, hard-magnetic soft beams, and complex shape-morphing structures. Subsequently, Garcia-Gonzalez [45] developed a continuum framework for modeling the effect of viscoelasticity on the magneto-mechanical response of hard-magnetic soft materials under dynamic and static loading conditions. Further, Ye et al. [46] developed a computationally efficient numerical model for hard-magnetic soft materials by decomposing the elastic deformation energy into lattice volumetric changes and stretching. Chen et al. [47] reported a theoretical model for characterizing the complex transformations of planar hard-magnetic soft beams. Chen et al. [48] developed a three dimensional theoretical model for analysing the extremely large deformations of hard-magnetic soft beams and provided the guidelines for the design and optimization of hard-magnetic soft structures. Considering the exact geometric nonlinearity, Chen et al. [49] developed a theoretical framework for predicting the magneto-mechanical response of functionally graded hard-magnetic soft beams. Zhang et al. [50] reported a micromechanics-based theoretical model for investigating the effect of interactions between the hard magnetic particles and the soft elastomer on the actuation performance of hard-magnetic soft materials. Considering visco-elastic effects, Dadgar-Rad and Hossain [51] reported a theoretical framework for the finite deformation analysis of hard-magnetic soft beam-type actuators subjected to magnetic loading. Kadapa and Hossain [52] developed a unified finite element model for the analysis of soft and hard magneto-active elastomers considering the time-dependent visco-elastic effects and simulated a magnetically driven four-finger robotic gripper. Dadgar-Rad and Hossain [53] developed a micropolar continuum theory and a finite element-based numerical model for the analysis of hard-magnetic soft materials. Further, a detailed review on fabrication, characterization, modeling, and applications of hard-magnetic soft materials can be found in Ref. [54].

The aforementioned theoretical studies expound on the analysis of hard-magnetic soft material-based actuators under quasi-statically applied magnetic fields. However, in many applications, specifically in soft robotics, hard-magnetic soft actuators undergo dynamic motions during their operation, and further, the actuators are also expected to attain accurate and fast positioning. The dynamic modeling and analysis of hard-magnetic soft actuators under dynamic modes of actuation are rarely explored [43]. In this context, very recently, Xing and Yong [43] developed an analytical model for analysing the non-linear dynamic response of a planar hard-magnetic soft actuator under DC dynamic and AC dynamic modes of actuation. The developed model did not account for the material damping and strain-stiffening effects. Further, in many applications of hard-magnetic soft actuators, it is also demanded that the actuator achieve the desired position quickly and accurately. Hence, it is important to design and develop a control strategy that can align the the actual response of the hard-magnetic soft actuator close to the desired response. To this end, the aim of the present work is to develop a dynamic model and an efficient control strategy considering the material damping and strain stiffening effects for the fast and accurate alignment of the transient response of the actuator to the desired position without residual vibrations.

The outline of the remainder of this article is as follows. In Section 2, we introduce the statement of the problem along with the development of a dynamic model for hard-magnetic soft actuators using the principle of the least action-based Euler–Lagrange equation of motion. For a hard-magnetic soft actuator driven by multi-step magnetic signals, an input-shaping technique for alleviating the residual vibrations is developed in Section 3. In Section 4, a thorough parametric investigation is performed for demonstrating the capability of the proposed technique to alleviate the residual vibrations. Finally, in Section 5, the salient concluding inferences drawn from the current study are summarized.

## 2. Problem Description and Dynamic Modeling

**Figure 1:**
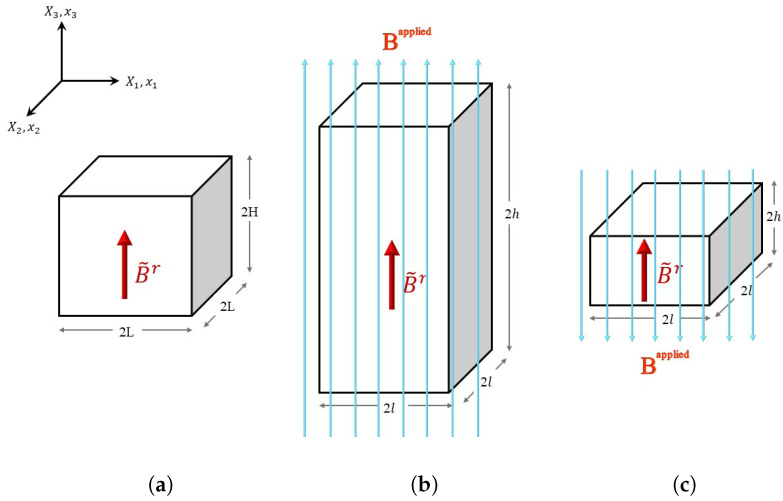
The schematic of a planar hard-magnetic soft actuator, (**a**) in the reference configuration with the dimensions 2L×2L×2H in the $X_{1}$, $X_{2}$, and $X_{3}$ directions, respectively; (**b**) in the current configuration with the dimensions 2l×2l×2h in the $x_{1}$, $x_{2}$, and $x_{3}$ directions, respectively, when the direction of the applied magnetic flux density is along the positive $x_{3}$ direction; and (**c**) in the current configuration with the dimensions 2l×2l×2h in the $x_{1}$, $x_{2}$, and $x_{3}$ directions, respectively, when the direction of the applied magnetic flux density is along the negative $x_{3}$ direction.

As shown in Figure 1, we consider a typical model of a planar actuator made up of an incompressible hard magnetic soft material. The coordinates $\left[x_{1},x_{2},x_{3}\right]$ denote the spatial points in the current configuration corresponding to the material points denoted by $\left[X_{1},X_{2},X_{3}\right]$ in the reference configuration. The actuator has size 2L×2L×2H in the reference/undeformed configuration [Figure 1a]. The direction of the residual magnetic flux density vector ${\tilde{B}}^{r}$ is considered to be along the positive $X_{3}$ direction in the undeformed configuration. When the direction of the applied magnetic flux density vector $B^{\mathrm{applied}}$ is along the positive $x_{3}$ axis, actuator expands as depicted in Figure 1b. The actuator contracts when the direction of the applied magnetic flux density vector $B^{\mathrm{applied}}$ is along the negative $x_{3}$ axis, as shown in Figure 1c. In the current/deformed configuration (Figure 1b,c), the actuator has dimensions 2l×2l×2h. Assuming the principal stretch along the $x_{3}$ direction to be $\lambda \left(t\right)=\frac{2h}{2H}$, and implementing incomprehensibility and symmetric geometry conditions [43], the following relationship is established between the current and reference coordinates of a hard-magnetic soft actuator:

(1) $$x_{1}=\frac{X_{1}}{\sqrt{\lambda (t)}},x_{2}=\frac{X_{2}}{\sqrt{\lambda (t)}},x_{3}=\lambda \left(t\right)X_{3},$$

and the corresponding deformation gradient tensor F is expressed as

(2) $$F=\left[\begin{array}{ccc} \frac{1}{\sqrt{\lambda (t)}} & 0 & 0\\ 0 & \frac{1}{\sqrt{\lambda (t)}} & 0\\ 0 & 0 & \lambda (t) \end{array}\right].$$

For modeling the magneto-mechanical behaviour of the hard-magnetic soft actuator, the constitutive model of an ideal hard-magnetic soft material given by Zhao et al. [44] is adopted. The constitutive model assumes that the residual magnetic flux density of an ideal hard-magnetic soft material will be constant as long as the hard magnetic particles remain magnetically saturated. Thus, the thermodynamics of an ideal hard-magnetic soft material are characterized by the Helmholtz free-energy density function, written as

(3) $$\psi =\psi^{\mathrm{elastic}}\left(F\right)-\frac{F{\tilde{B}}^{r}\cdot B^{\mathrm{applied}}}{\mu_{0}}$$

in which $\mu_{0}$ is the vacuum permeability, and $\psi^{\mathrm{elastic}}$ is the elastic energy density of the hard-magnetic soft material in the deformed state. In the current investigation, for accounting the effect of strain stiffening or polymer chain extensibility in hard-magnetic soft materials, the phenomenological Gent model of hyperelasticity [52,55] is considered to specify the elastic strain energy density function as

(4) $$\psi^{\mathrm{elastic}}=-\frac{GJ_{\lim}}{2}\ln\left[1-\frac{\left(I_{1}-3\right)}{J_{\lim}}\right],$$

where $I_{1}=\mathrm{tr}\left(F^{T}F\right)$, *G* is the shear modulus, and $J_{\lim}$ is a dimensionless material parameter accounting for the extensibility of polymer chains. Substituting the expressions for elastic strain energy density from Equation (4) and deformation gradient from Equation (2) into Equation (3), the free energy density function for the hard-magnetic soft material is written in terms of stretch parameter as [43,44]

(5) $$\psi =-\frac{GJ_{lim}}{2}\ln\left[1-\frac{\left(2\lambda^{-1}+\lambda^{2}-3\right)}{J_{\lim}}\right]-\frac{\lambda {\tilde{B}}^{r}B^{\mathrm{applied}}}{\mu_{0}}.$$

Next, we devise the governing dynamic equation of motion for a hard-magnetic soft actuator using the Euler–Lagrange equation [56]

(6) $$\frac{d}{dt}\left(\frac{\partial L}{\partial \dot{\lambda }}\right)-\frac{\partial L}{\partial \lambda }+\frac{\partial D}{\partial \dot{\lambda }}=0,$$

in which L is the Lagrangian and is defined as the difference of the kinetic energy *T* and potential energy *U* of the actuator, D represents the energy dissipation function, and $\dot{\lambda }$ denotes the time derivative of the principal stretch λ. The kinetic energy (T) of the hard-magnetic soft actuator is obtained as [43,57]

(7) $$T=\int_{\Omega }\frac{1}{2}\rho \left({{\dot{x}}_{1}}^{2}+{{\dot{x}}_{2}}^{2}+{{\dot{x}}_{3}}^{2}\right)d\Omega =\frac{2}{3}\rho HL^{4}{\dot{\lambda }}^{2}\lambda^{-3}+\frac{4}{3}\rho L^{2}H^{3}{\dot{\lambda }}^{2},$$

where ρ is the density of the hard-magnetic soft material and is constant throughout the deformation due to the incomprehensibility constraint, and Ω is the domain occupied by the deformed configuration. The total potential energy (U) of the actuator is obtained by multiplying the free energy density function (Equation (5)) with the actuator volume in the deformed configuration as

(8) $$U=-8HL^{2}\left[\frac{GJ_{\lim}}{2}\ln\left\{1-\frac{\left(2\lambda^{-1}+\lambda^{2}-3\right)}{J_{\lim}}\right\}+\frac{\lambda {\tilde{B}}^{r}B^{\mathrm{applied}}}{\mu_{0}}\right].$$

Hard-magnetic soft materials show damping effects during their operation due to material viscoelasticity [51,52]. Making an assumption that the damping forces are linear with respect to the velocity of deformation in the $x_{3}$ direction, the energy dissipation function D is written as [58,59]

(9) $$D=\frac{1}{2}c{\dot{\lambda }}^{2}H^{2},$$

where *c* is the damping coefficient. Substituting the expressions of total potential energy (U), kinetic energy (T), and energy dissipation function (D) from Equations (7)–(9) into the Euler–Lagrange Equation (6), the governing dynamic equation of the actuator is derived as

(10) $$\ddot{\lambda }-\frac{1.5{\dot{\lambda }}^{2}}{\lambda \left(1+c_{1}\lambda^{3}\right)}+\frac{6c\dot{\lambda }H\lambda^{3}}{8\rho L^{4}\left(1+c_{1}\lambda^{3}\right)}+\frac{6G}{\rho L^{2}\left(1+c_{1}\lambda^{3}\right)}\left[\frac{\lambda^{4}-\lambda }{\left\{1-\left(\frac{2\lambda^{-1}+\lambda^{2}-3}{J_{lim}}\right)\right\}}-\frac{{\tilde{B}}^{r}B^{applied}\lambda^{3}}{G\mu_{0}}\right]=0,$$

where $c_{1}=\frac{2H^{2}}{L^{2}}$ is a non-dimensional constant, and $\ddot{\lambda }$ is the second derivative of principal stretch λ with respect to time *t*. Further, defining the nondimensional time as $\tau =t\sqrt{\frac{G}{\rho L^{2}}}$, the nondimensional damping coefficient as $\xi =\frac{c}{8}\sqrt{\frac{1}{L^{2}H^{2}\rho G}}$, and the nondimensional magnetic flux density as $b=\sqrt{\frac{{\tilde{B}}^{r}B^{\mathrm{applied}}}{\mu_{0}G}}$, the governing dynamic equation (Equation (10)) is reduced to its nondimensional form as

(11) $$\ddot{\lambda }-\frac{1.5{\dot{\lambda }}^{2}}{\lambda \left(1+c_{1}\lambda^{3}\right)}+\frac{3\xi c_{1}\lambda^{3}\dot{\lambda }}{\left(1+c_{1}\lambda^{3}\right)}+\frac{6}{\left(1+c_{1}\lambda^{3}\right)}\left[\frac{\lambda^{4}-\lambda }{\left\{1-\left(\frac{2\lambda^{-1}+\lambda^{2}-3}{J_{\lim}}\right)\right\}}-b^{2}\lambda^{3}\right]=0,$$

in which $\dot{\lambda }$ and $\ddot{\lambda }$ are the first and the second derivatives of principal stretch λ with respect to nondimensional time τ. In this study, we assume that the actuator starts from rest and the corresponding initial conditions are given as

(12) $$\lambda =1,\;\dot{\lambda }=0.$$

**Figure 2:**
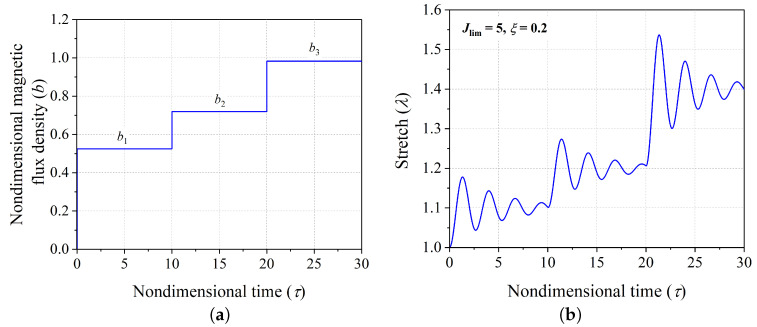
(**a**) A generic multi-step magnetic flux density input signal, and (**b**) the transient response of the hard-magnetic soft actuator.

The transient response of the actuator [stretch (λ) vs. nondimensional time (τ)] for any given magnetic loading function expressed in terms of a time-varying nondimensional magnetic flux density *b* can be obtained by numerically integrating the nondimensional governing dynamic equation (Equation (11)) along with the initial conditions (Equation (12)). In the context of defining the problem under consideration, Figure 2b shows illustrative response of the hard-magnetic soft actuator when actuated by the time varying multi-step nondimensional magnetic loading signal shown in Figure 2b. The polymer chain extensibility $J_{\lim}$ and nondimensional damping coefficient ξ are considered to be 5 and 0.2, respectively, for illustration. As evident from Figure 2b, the transient response of the actuator exhibits a significant oscillatory response about the respective steady state positions corresponding to the applied nondimensional magnetic flux density input signal, i.e., $b_{1}$, $b_{2}$, and $b_{3}$ [43]. In several application of hard-magnetic soft materials in the field of soft robotics, it is expected to achieve the desired equilibrium position or shift between the desired successive equilibrium positions with minimal residual oscillations, which creates the main motivation for the current investigation.

In the next section, we present the systematic design of a command shaping technique for alleviating the undesired oscillations associated with any desired equilibrium position.

## 3. Development of A Command-Shaping Technique for Alleviating the Residual Vibrations in Hard-Magnetic Soft Actuator

In this section, we propose an input/command-shaping technique for alleviating the residual oscillations in a planar hard-magnetic soft actuator subjected to the single-step and multi-step magnetic flux signals. The proposed control technique depends on setting the balance of different forces at the point of maximum stretch in a periodic vibration cycle.

**Figure 3:**
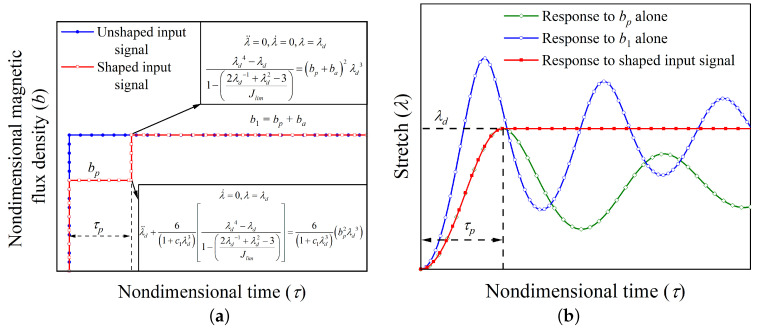
(**a**) Shaped and Unshaped magnetic flux density input signals showing the equations governing the actuator motion at two critical points, and (**b**) the transient response of the hard-magnetic soft actuator subjected to shaped and unshaped input signals.

Assume that the hard-magnetic soft actuator is expected to attain an equilibrium position defined by the stretch parameter equal to $\lambda_{d}$. In order to design an input magnetic flux density signal that satisfies the aforementioned requirement, firstly, by assigning zero value to the temporal terms in Equation (11), we obtain the magnitude of the quasi-statically applied nondimensional magnetic flux density $b_{1}$, which equilibrates the actuator at $\lambda \;=\;\lambda_{d}$. If the magnetic flux density $b_{1}$ is applied in the form of step input signal as represented in Figure 3a (labeled as an unshaped input signal), the planar actuator system vibrates about the chosen equilibrium position $\left(\lambda_{d}\right)$ as depicted in Figure 3b (labeled as a response to $b_{1}$ alone). To stabilize the actuator’s dynamic response, a two-step input signal is designed, wherein a lower magnetic flux density denoted as $b_{p}$ is applied in the form of a step signal for a dimensionless time period of $\tau_{p}$. These estimates of the applied step magnetic flux density $b_{p}$ and dimensionless time period $\tau_{p}$ are obtained by numerically integrating the governing equation (Equation (11)) in such a way that the maximum value of stretch parameter in the first cycle of vibration reaches the desired position $\lambda_{d}$ in time $\tau_{p}$. At this point with the maximum value of stretch parameter, the nondimensional stretch rate $\dot{\lambda }$ is zero, and the hard-magnetic soft actuator is acted upon by the nondimensional magnetic force $\left[\frac{6b_{p}^{2}\lambda_{d}^{3}}{\left(1+c_{1}\lambda_{d}^{3}\right)}\right]$, mechanical restoring force $\left[\frac{6(\lambda_{d}^{4}-\lambda_{d})}{\left(1+c_{1}\lambda_{d}^{3}\right)\left(1-\frac{2\lambda_{d}^{-1}+\lambda_{d}^{2}-3}{J_{\lim}}\right)}\right]$, and the inertial force $\left({\ddot{\lambda }}_{d}\right)$. The balance between these forces acted upon the actuator at the point with maximum stretch parameter is shown in Figure 3a. Under the action of these forces, the soft actuator will continue to vibrate until the mechanism of material damping brings the actuator to the static equilibrium position corresponding to the magnetic flux density $b_{p}$ (represented by the green line in Figure 3b). This occurs because of the lower value of the nondimensional magnetic force in comparison to the nondimensional mechanical restoring force at the point with the stretch value maximum, i.e., $b_{p}^{2}\lambda_{d}^{3}<\frac{\left(\lambda_{d}^{4}-\lambda_{d}\right)}{\left(1-\frac{2\lambda_{d}^{-1}+\lambda_{d}^{2}-3}{J_{\lim}}\right)}$. If the excess of the nondimensional mechanical restoring force is nullified by applying an additional nondimensional flux density step input signal of magnitude $b_{a}$, the actuator response gets stabilized at the desired equilibrium position. For keeping the actuator in static equilibrium at the desired equilibrium position $\lambda =\lambda_{d}$ without the inertial force, the addition step input magnetic flux density must satisfy the relation $b_{p}+b_{a}=b_{1}$. Thus, as shown in Figure 3a, an additional magnetic flux density $b_{a}$ is applied at nondimensional time $\tau =\tau_{p}$. The designed two-step input signal (command sequence) for achieving the desired equilibrium position is expressed mathematically as

(13) $$b\left(\tau \right)=\left\{\begin{array}{c} b_{p}\;\mathrm{for}\;0<\tau \leq \tau_{p}\\ b_{1}\;\mathrm{for}\;\tau_{p}<\tau \leq \tau_{1} \end{array}\right..$$

It is necessary to mention that, the governing dynamic Equation (11) being nonlinear, the values of intermediate nondimensional magnetic flux density $b_{p}$ and time $\tau_{p}$ are calculated numerically. The time-history response of the hard-magnetic soft actuator when subjected to the aforementioned two-step input signal (Equation (13)) is depicted in Figure 3b and designated as the response to shaped input. As evident from the Figure 3b, the shaped input response completely alleviates the existence of the residual vibrations.

**Figure 4:**
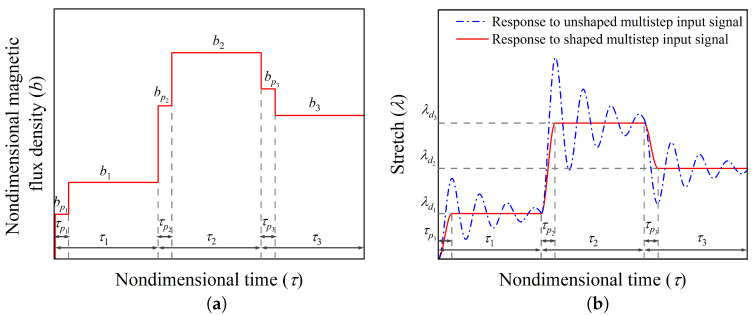
(**a**) Shaped multi-step magnetic flux density input signal for achieving three desired equilibrium positions, and (**b**) the transient response of the hard-magnetic soft actuator subjected to shaped and unshaped multi-step input signals.

Next, on the parallel lines of the input scheme developed for hard-magnetic soft actuators for achieving the desired equilibrium position without oscillation when subjected to a single-input signal, we designed a multi-step command shaping scheme (shown in Figure 4a) for achieving the equilibrium positions with stretch parameters $\lambda_{d1}$, $\lambda_{d2}$, $\lambda_{d3},\ldots \ldots \lambda_{dn}$, each lasting for $\tau_{1}$, $\tau_{2}$, $\tau_{3},\ldots \ldots \tau_{n}$ time spells, respectively. The associated equilibrium nondimensional magnetic flux density $b_{1}$, $b_{2}$, $b_{3}\ldots \ldots ,b_{n}$ is evaluated using Equation (11) by dropping all the time dependent terms. The intermediate values of the nondimensional magnetic flux density and corresponding nondimensional time ($b_{p1}$, $b_{p2}$, $b_{p3},\ldots \ldots b_{pn}$ and $\tau_{p1}$, $\tau_{p2}$, $\tau_{p3},\ldots \ldots \tau_{pn}$) are extracted numerically by solving the dynamic governing Equation (11). The resulting designed input signal is expressed mathematically as

(14) $$b\left(\tau \right)=\left\{\begin{array}{c} b_{p1}\;\mathrm{for}\;0<\tau \leq \tau_{p1}\\ b_{1}\;\mathrm{for}\;\tau_{p1}<\tau \leq \tau_{1}\\ b_{p2}\;\mathrm{for}\;\tau_{1}<\tau \leq \tau_{1}+\tau_{p2}\\ b_{2}\;\mathrm{for}\;\tau_{1}+\tau_{p2}<\tau \leq \tau_{1}+\tau_{2}\\ •\\ •\\ •\\ b_{pn}\;\mathrm{for}\;\sum_{k=1}^{n-1}\tau_{k}<\tau \leq \sum_{k=1}^{n-1}\tau_{k}+\tau_{pn}\\ b_{n}\;\mathrm{for}\;\sum_{k=1}^{n-1}\tau_{k}+\tau_{pn}<\tau \leq \sum_{k=1}^{n}\tau_{k} \end{array}\right..$$

From the response of the actuator shown in Figure 4b, it can be observed that the designed multi-step input signal [Figure 4a] significantly alleviates the residual vibrations about the desired position. In the upcoming section, we demonstrate the utility of the developed vibration control command/input shaping strategy by achieving different desired positions of the actuator and also investigate the effect of various parameters on the performance of the control scheme.

## 4. Results and Discussion

Based on the command shaping scheme explained in the previous section, this section explores the influence of various parameters such as the damping of the material, the extensibility of the polymer chains, and the sequence of desired equilibrium states on the alleviation of residual vibrations in a multi-step magnetically actuated hard-magnetic soft actuator.

**Figure 5:**
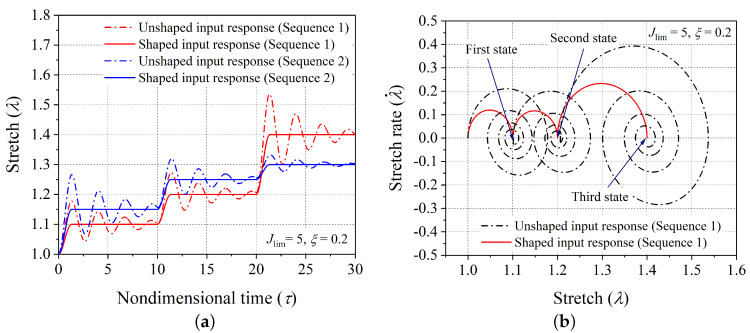
(**a**) Controlled and uncontrolled transient response for the two desired equilibrium sequences ((1.10, 10), (1.20, 10), (1.40, 10)) and ((1.15, 10), (1.25, 10), (1.35, 10)) of the hard-magnetic soft actuator, and (**b**) controlled and uncontrolled phase-plane portraits for the desired equilibrium sequence ((1.10, 10), (1.20, 10), (1.40, 10)).

**Figure 6:**
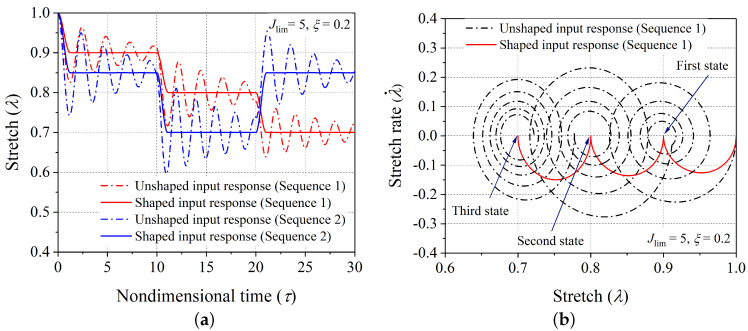
(**a**) The controlled transient response (corresponding to shaped input signal) and uncontrolled transient response (corresponding to unshaped input signal) for the two chosen equilibrium sequences ((0.90, 10), (0.80, 10), (0.70, 10)) and ((0.85, 10), (0.70, 10), (0.85, 10)) of the hard-magnetic soft actuator, and (**b**) the uncontrolled (corresponding to unshaped input signal) and controlled (corresponding to shaped input signal) phase−plane plots for the chosen ((0.90, 10), (0.80, 10), (0.70, 10)) equilibrium sequence.

Firstly, the validity of the proposed scheme of controlling vibrations is demonstrated through aligning the dynamic response of the hard-magnetic soft actuator (in both the modes: expansion (Figure 1b) and contraction (Figure 1c)) for two different sequences of desired equilibrium positions. For the expansion case of the hard-magnetic soft actuator, the two equilibrium sequences ((1.10, 10), (1.20, 10), (1.40, 10)) and ((1.15, 10), (1.25, 10), (1.35, 10)) are considered. Here, in each component of the equilibrium sequence, the first number shows the stretch parameter $\left(\lambda_{d}\right)$ corresponding to the chosen equilibrium position of the actuator, and the second number represents the span of the nondimensional time for which that equilibrium position should end. If the actuator is subjected to the magnetic loading applied through a series of three step signals, with each of them having the steady state solution as the desired actuator position, the resulting dynamic response will inherently vibrate about the desired equilibrium stretch level. Such uncontrolled dynamic responses (when subjected to unshaped input signal) of the actuator (corresponding to the aforementioned equilibrium sequences) are shown in Figure 5a and labeled as an unshaped input response. If the time span of any equilibrium sequence is large enough, the material damping will gradually bring the uncontrolled dynamic response of the actuator to the chosen equilibrium position of the actuator. However, the developed input shaping control scheme gives quick shifting between the chosen equilibrium positions of the hard-magnetic soft actuator without any residual vibrations. But, as depicted in Figure 4a, the shaped input signal (corresponding to a controlled response) has six steps in comparison with the unshaped input signal (corresponding to a controlled response), which has only three steps. Further, the controlled dynamic response of the hard-magnetic soft actuator, when subjected to the designed six step input signal, is plotted in Figure 5a for the aforementioned two equilibrium sequences and labeled as a shaped input response. For the first equilibrium sequence ((1.10, 10), (1.20, 10), (1.40, 10)), the phase-plane plots corresponding to the applied shaped and unshaped input signals are shown in Figure 5b. From the phase-plane plots, it is observed that when the hard-magnetic soft actuator is subjected to the unshaped three-step input signal, the actuator exhibits residual vibrations (periodic orbits) about each chosen equilibrium position (designated by • symbol). However, as expected, when the actuator is subjected to the shaped six-step input magnetic loading, the actuator moves to the required position without vibrations. In parallel lines, we assessed the performance of the developed control scheme in suppressing the residual oscillation exhibited by the hard-magnetic soft actuator in the contraction mode of actuation (Figure 1c) by considering ((0.90, 10), (0.80, 10), (0.70, 10)) and ((0.85, 10), (0.70, 10), (0.85, 10)) equilibrium sequences. For these sequences, the uncontrolled and controlled dynamic responses and the phase-plane plots are depicted in Figure 6a and Figure 6b, respectively. From Figure 5 and Figure 6, it is inferred that the proposed input-shaping scheme can be used for controlling the vibrations in a hard-magnetic soft actuator for any user-chosen steady-state position, and this ascertains the efficacy of the developed technique.

**Figure 7:**
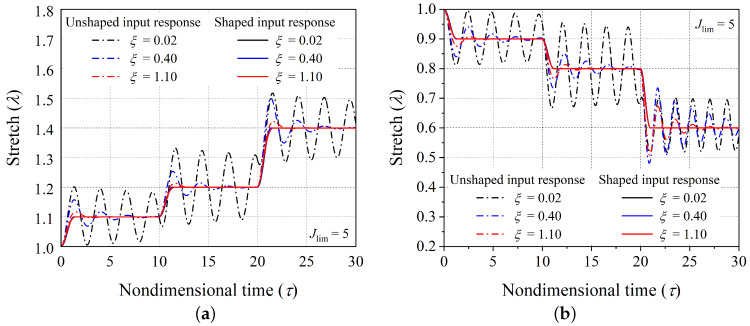
The controlled transient response (corresponding to shaped input signal) and uncontrolled transient response (corresponding to unshaped input signal) of the hard-magnetic soft actuator for different values of nondimensional damping coefficient ξ, in the (**a**) expansion mode of operation, and in the (**b**) contraction mode of operation.

**Figure 8:**
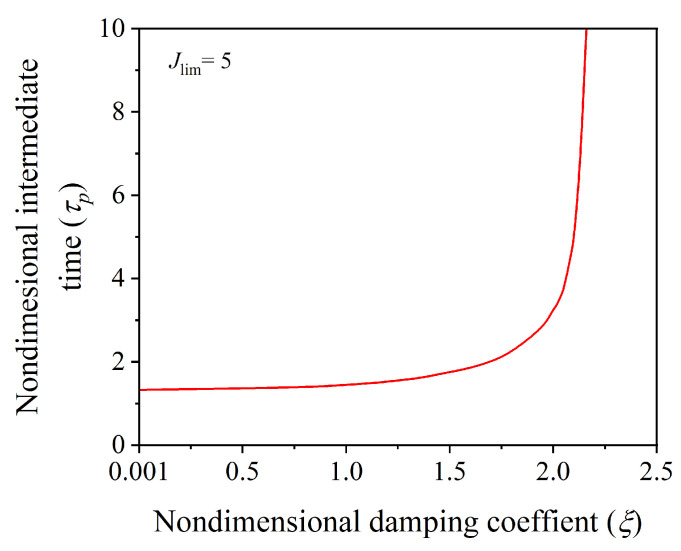
Effect of material damping on the nondimensional intermediate time ($\tau_{p}$) required for attaining the desired equilibrium position.

Next, we investigate how the material damping affects the control of the residual vibrations. For this investigation, We consider cases with different values of the nondimensional material damping coefficient ξ=0.02,0.40,and1.10. Figure 7a,b shows the effect of material damping on the controlled dynamic response of the hard-magnetic soft actuator in expansion mode of actuation with the equilibrium sequence ((1.10, 10)], (1.20, 10), (1.40, 10)) and in the contraction mode of actuation with the equilibrium sequence ((0.90, 10), (0.80, 10), (0.60, 10)), respectively. In both the modes of operation, it is observed from Figure 7 that the uncontrolled response of the actuator with a high value of the damping coefficient reaches the equilibrium position very quickly, and vice versa. Further, it is also evident from Figure 7 that the material damping has very little impact on the controlled dynamic response of the actuator, showing that the developed input-shaping control technique can take in a wide span of viscous coefficients of damping specific to many potential uses of hard-magnetic soft materials. The change in the nondimensional intermediate time $\left(\tau_{p}\right)$ taken by the hard-magnetic soft actuator in reaching the desired equilibrium position ($\lambda_{d}=1.1$) as a function of the nondimensional damping coefficient is shown in Figure 8. From Figure 8, it is observed that the intermediate time is strongly dependent on the nondimensional damping coefficient in the neighbourhood of a limiting case, as the damping coefficient reaches 2.14. This represents the situation of a critically damped system.

**Figure 9:**
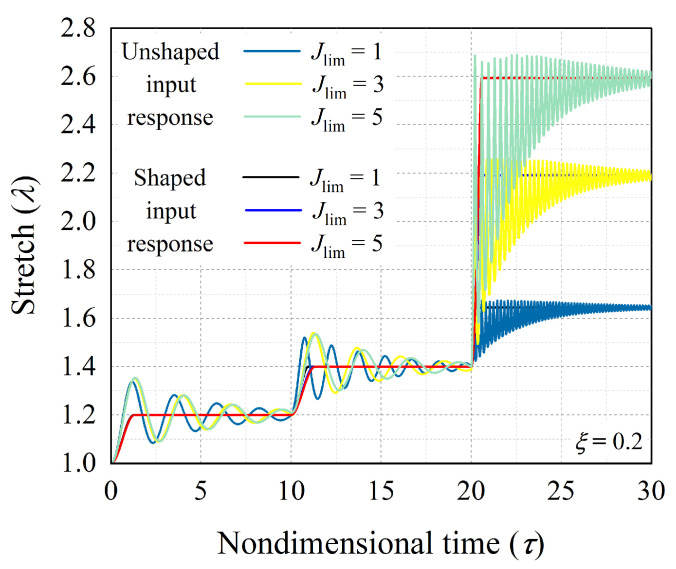
The controlled transient response (corresponding to shaped input signal) and uncontrolled transient response (corresponding to unshaped input signal) of the hard-magnetic soft actuator for different values of $J_{\lim}$ (polymer chain extensibility parameter).

**Figure 10:**
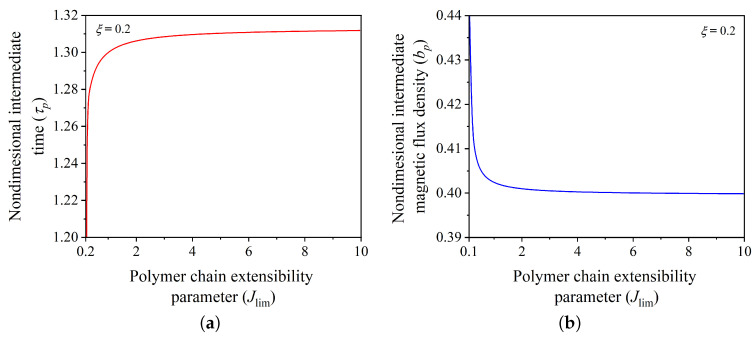
Variation of (**a**) nondimensional intermediate time ($\tau_{p}$) and (**b**) nondimensional intermediate magnetic flux density ($b_{p}$) required by the hard-magnetic soft actuator to attain the desired equilibrium position as a function of extensibility of polymer chains $\left(J_{\lim}\right)$.

The polymer chains of soft elastomers or polymeric materials have limiting length, which limits the deformation of the elastomer during extension [8,21,52]. Next, we investigated the effect of polymer chain extensibility on the control of dynamic response of hard-magnetic soft actuator. Here, in the adopted material model (Equation (5), the limiting stretch effect is governed by the parameter $J_{\lim}$. We considered three different levels of polymer chain extensibility $J_{\lim}=1,3,\mathrm{and}5$. For these different values of $J_{\lim}$ and and non-dimensional damping coefficient ξ=0.2, Figure 9 shows the effect of polymer chain extensibility on the uncontrolled and controlled dynamic responses of the hard-magnetic soft actuator for achieving the equilibrium positions represented by the sequence ((1.2, 10), (1.4, 10), (${\tilde{\lambda }}_{m}$, 10)), in which ${\tilde{\lambda }}_{m}$ represents the stretch near the limiting stretch $\lambda_{m}$, obtained from equation $\frac{2}{\lambda_{m}}+\lambda_{m}^{2}-3=J_{\lim}$. For the considered three different levels of $J_{\lim}=1,3,\mathrm{and}5$, the limiting stretch is equal to 1.675, 2.262, and 2.694, respectively. It is evident from Figure 9 that the number of oscillation cycles exhibited by the actuator before stabilizing to the equilibrium state near the limiting stretch are very large in comparison with the other two states. From Figure 9 it can also be observed that the developed command-shaping strategy efficiently controls the large actuation/deformation response $\left(\lambda =\lambda_{m}\right)$ of the system. Figure 10a,b shows the change in the nondimensional intermediate time $\left(\tau_{p}\right)$ and the nondimensional magnetic flux density $\left(b_{p}\right)$ required by the actuator to achieve the first equilibrium position in aforementioned sequence as a function of polymer chain entanglement parameter $J_{\lim}$. Figure 10 suggests that the time taken by the actuator in switching between any two equilibrium states increases with increasing polymer chain extensibility. In contrast, the magnetic flux density required for achieving a desired equilibrium position decreases with increasing polymer chain extensibility, demonstrating a favorable influence on the actuator response. In the following section, we provide the summary of the salient conclusions drawn from the current study.

## 5. Conclusions

Magnetically driven hard-magnetic soft actuators operating in the dynamic mode (driven by a multi-step input signal) exhibit inherent residual oscillations before attaining the desired equilibrium positions. It is crucial to mitigate such inherent residual oscillations about the desired steady state to enhance the positional accuracy and the transit time between two successive equilibrium positions. To mitigate such residual vibrations, this paper reported an input-shaping technique that relies on establishing a balance between the mechanical and magnetic forces at the point of maximum stretch in an oscillation cycle. Using the Euler–Lagrange equation of motion and considering the strain stiffening effects, the equation governing the dynamics of the hard-magnetic soft actuator is derived for simulating the controlled and uncontrolled dynamic responses. A parametric study is carried out for demonstrating how the extensibility of polymer chains, sequence of desired equilibrium positions, and material damping affect the performance of the developed input-shaping scheme. In case of attaining any desired equilibrium state, the designed shaped input magnetic flux density signal is not affected significantly by variations in the material damping in the hard-magnetic soft material. A higher level of polymer chain extensibility in hard-magnetic soft material results in increasing the time taken by the actuator to attain the desired steady state/equilibrium position. However, there is a concomitant reduction in the required magnetic flux density, showing a favorable influence of the extensibility of polymer chains on the performance of the actuator. The devised control scheme and the conclusions can be potentially useful in designing an open-loop controller for magnetically driven hard-magnetic soft material-based systems.

In the present work, we considered an idealized model of the hard-magnetic soft actuator and placed more emphasis on the underlying mechanics to design the input shaping scheme for alleviating system vibrations. The developed dynamic model and input shaping scheme can be further extended to account for the effect of visco-elasticity, polymer chain entanglements and cross-links, temperature, compressibility of hard-magnetic soft materials, etc. Further experimental work would be necessary to corroborate the input-shaping control scheme developed in the present work.

## Data Availability

The data presented in this study are available on request from the corresponding author.

## Nomenclature

$\left[x_{1},x_{2},x_{3}\right]$	Spatial coordinates
$\left[X_{1},X_{2},X_{3}\right]$	Material coordinates
2H	Thickness of the actuator in the reference configuration
2L	Length and breadth of the actuator in the reference configuration
2h	Thickness of the actuator in the current configuration
2l	Length and breadth of the actuator in the current configuration
${\tilde{B}}^{r}$	Residual magnetic flux density vector
$B^{\mathrm{applied}}$	Applied magnetic flux density vector
λ(t)	Principal stretch along the $x_{3}$ direction
F	Deformation gradient tensor
ψ	Helmholtz free-energy density function
$\mu_{0},\;G,\;J_{\lim},\;\rho$	Material parameters
*c*	Damping coefficient
L	Lagrangian
*t*	Time
*T*	Kinetic energy
*U*	Total potential energy
D	Energy dissipation function
$\dot{\lambda }$	Time derivative of the principal stretch
τ	Nondimensional time
ξ	Nondimensional damping coefficient
b	Nondimensional magnetic flux density
$c_{1}$	Nondimensional constant
Ω	Deformed configuration of the actuator
